# Changes in the prevalence of obesity in Czech adolescents between 2018 and 2022 and its current non-genetic correlates – HBSC study

**DOI:** 10.1186/s12889-023-17010-x

**Published:** 2023-10-25

**Authors:** Erik Sigmund, Dagmar Sigmundová, Jan Pavelka, Michal Kalman, Jaroslava Voráčová, Zdeněk Meier, Jaroslava Kopčáková, Petr Badura

**Affiliations:** 1 Faculty of Physical Culture, Institute of Active Lifestyle, Faculty of Physical Culture, Palacký University Olomouc, Olomouc, 771 11 Czech Republic; 2 Faculty of Physical Culture, Department of Recreation and Leisure Studies, Palacký University Olomouc, Olomouc, 771 11 Czech Republic; 3 Faculty of Physical Culture, Department of Social Sciences in Kinanthropology, Palacký University Olomouc, Olomouc, 771 11 Czech Republic; 4 Sts Cyril and Methodius Faculty of Theology, Social Health Institute, Palacký University Olomouc, Olomouc, 771 11 Czech Republic; 5 Department of Health Psychology and Research Methodology, Faculty of Medicine, P.J.Safarik University, Kosice, 040 01 Slovakia

**Keywords:** Obesity, Physical activity, Eating habits, Sleeping, Socioeconomic status, HBSC study

## Abstract

**Background:**

The main aim of the study is to examine changes in the prevalence of obesity in Czech adolescents between 2018 and 2022 and its current non-genetic correlates with respect to the adolescents’ families’socioeconomic status (SES) in 2022.

**Methods:**

The sample of 24,535 adolescents (n = 11,629/12,906_2018/2022_; boys: 50.4/50.6%_2018/2022_) aged 10.5–16.5 years that was analysed was drawn from two nationally representative cohorts of Czech youngsters from the last two cycles of the Health Behaviour in School-aged Children (HBSC) online questionnaire survey from 2018 to 2022. Obesity is represented by the > 97th percentile interval on the World Health Organization Body Mass Index percentile chart, with distinctions by sex and the age of adolescents. The differences in the prevalence of obesity between boys and girls from all SES family categories in 2018 and 2022 were tested using a chi-square test (*χ*^2^). Multiple logistic regression analysis with repeated measures was used to analyse correlates of obesity in 2022.

**Results:**

Between 2018 and 2022, there was no significant difference in the prevalence of obesity in girls or boys in any of the SES categories of families. Adolescents from low-SES families have the highest prevalence of obesity, 11% for boys and 5.8% for girls, significantly higher (p < .001) than its prevalence among adolescents from high-SES families, by + 4.8% points for boys and + 3.9% points for girls. Among adolescents from low-SES families, individuals who engaged in moderate-to-vigorous physical activity (PA) daily (p < .005) or vigorous PA three times per week (p < .05) were significantly less likely to be obese than their less active peers. Skipping breakfast significantly (p < .05) increased the odds of obesity, but only among adolescents from low-SES families. Shorter screen time (ST) significantly (p < .05) reduced the odds of obesity for all categories of adolescent SES.

**Conclusions:**

Obesity is most pronounced in adolescents from low-SES families as a result of a long-term positive energy balance mediated by unbalanced behaviour. Significantly lower odds of obesity in adolescents from low-SES families were confirmed to be associated with regular practice of the recommended PA, shorter ST, and not skipping breakfast.

## Background

The high prevalence of obesity across the age spectrum of the population continues to be a major health concern [1] and there is a societal need to provide current valid information on changes in the prevalence of obesity and its correlates found in children, adolescents, adults, and the elderly. Because genetic factors alone cannot fully explain the obesity pandemic in children and adolescents [2], it is important to recognise that obesity is primarily caused by a long-term positive energy balance between energy intake and energy expenditure that is mediated by a complex interaction of multiple psychosocial and biological correlates and family conditions [2, 3]. Preventive factors for obesity include adequate levels of daily moderate-to-vigorous physical activity (MVPA) and adequate sleep, while excessive television, computer, and mobile device viewing (referred to collectively as screen time (ST)), insufficient sleep, and regular consumption of sugar-sweetened beverages are considered to represent unhealthy behaviours that increase the incidence of obesity [3–5]. However, the prevalence of obesity and its correlates can change significantly as individuals’ living conditions change, and such dramatic changes have been brought about by the COVID-19 pandemic and the measures taken to contain it [6, 7]. Apart from the disrupted school regime, the content of out-of-school time during the COVID-19 pandemic was also different from normal. Organised sports activities were prohibited both indoors and outdoors [8], and opportunities for direct interaction with peers were very limited [9]. Overall, for adolescents, this meant that their daily routines, in which school and organised leisure activities occupied a significant portion of their time, disappeared [10].

Both trend [11, 12] and systematic review [13] studies have repeatedly found a higher prevalence of obesity in adolescents from low-SES families than in adolescents from high-SES families from Canada, the United States, and Australia. Moreover, the difference in the prevalence of obesity between adolescents from low-SES versus high-SES families has been increasing steadily, and this was the case even in pre-pandemic times [11, 12]. A similar trend of the widening of the gap in the prevalence of obesity between adolescents from low- and high-SES families was observed between 2010 and 2018 in 11-15-year-old boys and 2014 and 2018 in 11-15-year-old girls from the Czech Republic [14]. However, the future direction of the prevalence of obesity among Czech adolescents remains unknown. Available studies show that during the COVID-19 pandemic, children and adolescents were at significantly increased risk of developing obesity or worsening obesity-related diseases [6, 7], and the prevalence of obesity in adolescents in the United States, China, Italy, Spain, Chile, Brazil, Colombia, and Palestine increased over that period [6].

This rightly reinforces the call for changes in health policy in the post-COVID-19 era that would focus on more vigorous and effective reduction of obesity through changing the lives of children and adolescents. Implementing changes in the living conditions of children and adolescents emphasises the necessary collaboration among parents, physicians, coaches, policy makers, and all the co-creators of young people’s daily lifestyles [1, 6, 7]. However, the development of effective obesity reduction strategies and programmes is contingent on the most accurate information about the current prevalence of obesity and its correlates that can be influenced and changed. Therefore, the present study seeks to contribute to uncovering changes in lifestyle and health indicators in adolescents after the passing of the the COVID-19 pandemic by describing current changes in the prevalence of adolescent obesity and its correlates. The main objective of the study is to identify changes in the prevalence of obesity in Czech adolescents between 2018 and 2022 and its current non-genetic correlates with respect to adolescents’ families’ SES in 2022.

## Methods

### Study design

The comparative study is based on two consecutive cycles of cross-sectional online data collection from the Health Behaviour in School-aged Children (HBSC) study. The international HBSC study is conducted simultaneously in more than 50 countries under the auspices of the World Health Organization (WHO). Its mission is to provide a wide range of professionals, national and regional policy makers, educators, and social workers with relevant, up-to-date data on adolescents’ health and well-being in their social contexts at school, in the family, among friends, and in the neighbourhood, in order to explain and subsequently improve adolescents’ lifestyles and health [15, 16]. The HBSC is conducted using a standardised, internationally developed research protocol containing a self-assessment questionnaire that is administered simultaneously in all participating countries for cohorts of 11-, 13-, and 15-year-old adolescents to establish consistency in the data collection and processing process [17, 18]. To ensure international comparability, the core self-assessment questionnaire contains the same mandatory question section followed by optional question modules chosen by each country. All the questions of the self-assessment questionnaire are continuously developed and validated. The final form of the questionnaire used contains only validated questions with a clear and unambiguous data tracking procedure [17, 18]. As the HBSC study is focused on school-aged adolescents, in the Czech Republic data collection is conducted via an online form directly in schools.

### Participants and procedure of data collection

The sample of 24,535 adolescents (n = 11,629/12,906_2018/2022_; boys: 50.4/50.6%_2018/2022_) aged 10.5–16.5 years that was analysed was drawn from two nationally representative samples of Czech youngsters from the last two cycles of the HBSC questionnaire survey in 2018 and 2022, obtained through multistage stratified sampling by region, school type (ratio of primary schools to multi-year grammar schools), and school size (Table 1).

**Table 1:** Descriptive characteristics of the samples, HBSC study, Czech Republic 2018–2022†

	2018		2022	
	Boys	Girls	Boys	Girls
n=	(5856)	(5773)	(6532)	(6374)
	**%**	**%**	**%**	**%**
**Age category** ^§^				
11 years	32.7	32.8	33.3	34.3
13 years	34.5	34.4	35.1	34.3
15 years	32.8	32.8	31.6	31.4
**SES**				
Low	23.7	27.2	21.0	22.8
Medium	45.3	44.6	46.7	47.9
High	31.0	28.2	32.3	29.3
Weight status*				
Non-overweight	73.3	84.7	71.8	83.9
95% CI	72.1–74.4	83.8–85.7	70.6–72.9	83.0-84.9
Overweight	17.9	11.9	19.7	12.7
95% CI	16.9–19.0	11.0-12.7	18.7–20.7	11.8–13.6
Obesity	8.8	3.4	8.5	3.4
95% CI	8.1–9.6	2.9–3.9	7.8–9.2	2.9–3.8

*n* number of participants; ^§ 1^11 years (13 years and 15 years) includes adolescents in the age range 10.5-12.49 years (12.50-14.49 years and 14.50-16.49 years); †the weights for strata (the number of pupils/students in given grades in each region of Czech Republic) were applied; *SES* socioeconomic status; *obesity and overweight were represented by the > 97th percentile and 85th -97th percentile, respectively, on gender-specific Body Mass Index-for-age growth charts [19, 20]; *CI* 95% confidence interval

The primary sampling unit in both data collection cycles was the school class. Subsequently, one class from each school was randomly selected from grades 5, 7, and 9 (or from the corresponding grade in multi-year grammar schools). The data collection took place in the spring months of 2018 and 2022. Response rates ranged from 86 to 97% at the school level and exceeded 83–86% for pupils and students in both data collection cycles.

A trained team of researchers moderated the online data collection during a one-hour session in the school IT classroom following a presentation of the research. Prior to the presentation of the research and instructions for completing the questionnaire, the participants were assured of the voluntary and anonymous nature of their participation. The participants did not give their name anywhere and could discontinue their participation in the research at any time, refuse completely, or skip questions they were uncomfortable with. The parents/guardians of the adolescents were informed about the study through the school management and could opt their children out if they did not consent to their participation. Before completing the questionnaire, the participants confirmed their informed consent to participation in the research. The study design and methodology were approved by the Institutional Ethics Committee of the Faculty of Physical Culture of Palacký University in Olomouc with the reference numbers 9/2016 for the 2018 data collection and 65/2020 for the 2022 data collection.

### Survey items

#### Dependent variable – obesity

The variables of chronological age (years, months), body weight (kg), and height (cm) given on the current date of completing the HBSC questionnaire were used to calculate the weight status of the adolescents. The Body Mass Index (BMI) was calculated as the ratio of body weight (kg) to the square of body height (cm). The body weight level of the adolescents (non-overweight, overweight, obese) was derived using the WHO’s gender-specific BMI-for-age growth charts. Obesity and overweight were represented by the > 97th percentile and 85th -97th percentile, respectively, on the gender-specific BMI-for-age growth charts [19, 20] (Table 1). High correlations between self-reported anthropometric data (body weight, body height and calculated BMI) and subsequently objectively measured anthropometric data by researchers were revealed in a cross-sectional study of 10-15-year-old school children from Switzerland (body weight r = .96; body height r = .92; BMI r = .88) [21]. Self-reported values of body weight and calculated BMI were slightly overestimated, and height was slightly underestimated compared to objectively measured variables. Self-reported values tend to be more reliable in adolescents older than 11 years [21]. Therefore, self-reported anthropometric variables remain an appropriate choice to identify excessive body weight in 11-year-old and older adolescents in epidemiological studies [21, 22].

#### SES as a determinant of obesity

Previous studies have repeatedly found differences in the prevalence of obesity according to family SES in children and adolescents in a national context [1, 11, 12, 14] as well as in international comparisons [13, 16]. Therefore, statistical analyses will be conducted separately according to the SES of the participants’ families. The HBSC study uses the Family Affluence Scale (FAS) [16, 18], which has been validated as a valid indicator of relative wealth [23, 24] to identify low- and high-income households [25], to measure the SES of participants’ families. The FAS includes a six-item assessment of common material assets or activities involving the following: ownership of a car, van, or truck (responses: none, one, two or more); your own bedroom for yourself (none, yes); the number of family vacations/holidays abroad in the last year (not at all, once, twice, more than twice); the number of computers owned (none, one, two, more than two); ownership of a dishwasher (none, yes), and the number of bathrooms in the household (none, one, two, more than two) [16]. Responses were scored (none = 0; one/one = 1; two = 2; more than two = 3) and a summary score was created by summing all the FAS-related responses together. This FAS summary score was used to determine the SES of the families of the adolescents in the lowest 20% (low wealth), middle 60% (medium wealth), and highest 20% (high wealth) [16, 24]. In the socioeconomic conditions of Czech Republic, the FAS was validated in relation to the disposable household income (Pearson correlation r = .773 *p* < .001) [26].

#### Independent variables in energy balance-related behaviour

On the basis of the results of a previous trend study [14], the following variables were selected as potentially relevant correlates of obesity, representing the three dominant categories of an individual’s energy balance behaviour: (i) energy expenditure (daily MVPA, weekly vigorous physical activity (VPA), weekly participation in organised sport and daily ST), (ii) energy intake (daily consumption of sweets, daily breakfast) and (iii) sleep duration (daily sleep time).

The group of questions associated with energy expenditure is represented by the following four simple questions: frequency of MVPA for at least 60 min per day in the past seven days (responses ranged from zero days to seven days); frequency of exercise in free time that leads to shortness of breath or sweating (i.e. VPA) (none, less than once a month, once a month, once a week, twice a week, three times a week, four to six times a week, every day); participation in organised activities, individual and team sports run by sports clubs or other organisations (2018: no, yes; 2022: I don’t do this type of activity, once a month, once a week, twice or more a week); daily time spent using screen devices in free time (none at all, about half an hour a day, about one hour a day, about two hours a day, about three hours a day, about four hours a day, about five hours a day, about six hours a day, and about seven or more hours a day) [18]. For the statistical analyses, responses to PA-related questions were dichotomised according to the WHO guidelines as follows: MVPA ≥ 60 min per day vs. less frequent and VPA ≥ 3 days per week vs. less frequent [27]. The participating adolescents were categorised as ‘active’ (involved in organised team and/or individual sport) or ‘inactive’ (not involved in any organised sport). According to recent results focused on understanding the impact of technology on well-being, Czech adolescents spend on average four hours and 11 min per day on screen devices [28]. Therefore, the variable daily ST time was dichotomised as follows: ST ≥ 4 h per day vs. <4 h per day [28].

The self-reported MVPA and VPA assessments over the past seven days in 15-year-old adolescents were originally developed and validated against continuous measures using a Computer Science Application (CSA) accelerometer (r_MVPA_=0.40 *p* < .001; r_VPA_=0.36 *p* < .01) [29]. The participation in organised activities scale has an acceptable level of agreement (ICC = 0.64), indicating good reliability [30]. The acceptable seven-day stability of ST-related questions (television viewing (TV) and computer use (PC)) has been repeatedly verified in 11-15-year-old adolescents for weekdays (ICC_TV_=0.54–0.72 and ICC_PC_=0.33–0.82) and weekends (ICC_TV_=0.58–0.68 and ICC_PC_=0.33–0.66) [31–34]. A recent systematic review study confirms that the HBSC questionnaire items on PA and ST are reliable in assessing PA and sedentary behaviour in adolescents [35].

For the present study, two variables were selected from the HBSC questionnaire that relate to an individual’s energy intake: frequency of consumption of sweets (never; less than once a week; once a week; two to four times a week; five to six times a week; once a day; more than once a day), and regularity of daily breakfast on school days (never; one day; two days; three days; four days; five days) and weekend days (never; only on one day; on both days). The responses were recorded in a dichotomous outcome variable for the consumption of sweets (‘dailyʼ vs. ‘less than daily’) and a five-category outcome variable for regularity of breakfast: ‘daily’, ‘always at the weekend and occasionally during the week’, ‘daily on weekend days only’, ‘inconsistent’, and ‘never’.

Sleep time was calculated from the adolescents’ self-reported bedtime and waking-up times on school days and weekend days separately. The self-reported sleep time alternatives ranged in half-hour intervals from ‘no later than 9:00 p.m.’ to ‘2:00 a.m. or later’ for school days and ‘no later than 9:00 p.m.’ to ‘2:00 a.m. or later’ for weekend days. The response scale for waking-up times contained categories ranging in half-hour intervals from ‘no later than 5 a.m.’ to ‘8 a.m. or later’ for school days and ‘no later than 7 a.m.’ to ‘2 p.m. or later’ for weekends [36]. Finally, sleep time was calculated as the difference between bedtime and waking-up time, separately for weekdays and weekend days. In accordance with age-categorised sleep duration recommendations [37], sufficient sleep duration was defined as 9–12 h per 24 h for 11-year-old adolescents and 8–10 h per 24 h for 13- and 15-year-old adolescents. Daily sleep time shorter than the lower limit of the interval for sufficient sleep was categorized as insufficient sleep, and sleep time longer than the upper limit of the interval for sufficient sleep was categorized as excessive sleep. The sleep time variable entering the statistical analyses was derived from the cluster analysis according to the consensus age-related recommendation for hours of sleep [37] as follows: ‘sufficient at the weekend and during the week’ (Mean: M_weekend_/M_week_=9:41/8:05 h), ‘sufficient at the weekend, insufficient during the week’ (Mean: M_weekend_/M_week_=9:27/7:16 h), ‘excessive at the weekend and sufficient during the week’ (Mean: M_weekend_/M_week_=11:18/8:47 h), and ‘insufficient at the weekend and during the week’ (Mean: M_weekend_/M_week_=7:12/7:22 h). The HBSC sleep-time related questions show substantial reliability (ICC = 0.75/0.64 for bedtime on school days/weekend days; ICC = 0.77 for waking up on school days), and almost perfect reliability (ICC = 0.83) for the waking up at weekends item [32]. Moderate to strong criterion validity and the strong reliability of a self-reported sleep duration questionnaire have been repeatedly demonstrated in adolescents [38].

### Data analysis and statistical processing

After the raw data from the HBSC questionnaires from all school classes had been received, all processing and subsequent statistical processing was conducted in the Statistical Package for the Social Sciences (SPSS) for Windows v.28 software (IBM Corp. Released 2021. Armonk, NY, USA). Given the study objective, checks for nonsensical and incorrect responses or incomplete questionnaire completion were performed identically for the 2018 and 2022 datasets in accordance with the HBSC study methodological protocol [17, 18]. Percentages (%) supplemented with 95% confidence intervals (CIs) in the case of the body weight level category were used to describe the variables. Cluster analysis was used to categorise the amount of sleep on school days and at weekends in accordance with the recommendations for sleep in children and adolescents [37]. A chi-square (χ^2^) test was repeatedly used to test for differences in the prevalence of obesity by sex and SES category in adolescents between 2018 and 2022, as well as to determine differences associated with SES categories in the prevalence of obesity in 2022 separately for boys and girls. χ^2^ tests were also repeatedly used to analyse differences in selected correlates of obesity in relation to the adolescent SES categories and to test the statistical significance of differences in the prevalence of obesity by adolescent MVPA and VPA levels, participation in organised sports, ST level, and frequency of daily consumption of breakfast and sweets and sufficient sleep. A series of multivariate logistic regression analyses in the 2022 data collection were used to uncover the correlates of obesity separately for the adolescent SES categories. The results of the logistic regression analyses were expressed using odds ratios (ORs) and 95% confidence intervals (95% CIs). The alpha significance level was set at a minimum of 0.05.

## Results

### Changes in the prevalence of obesity

**Fig. 1:**
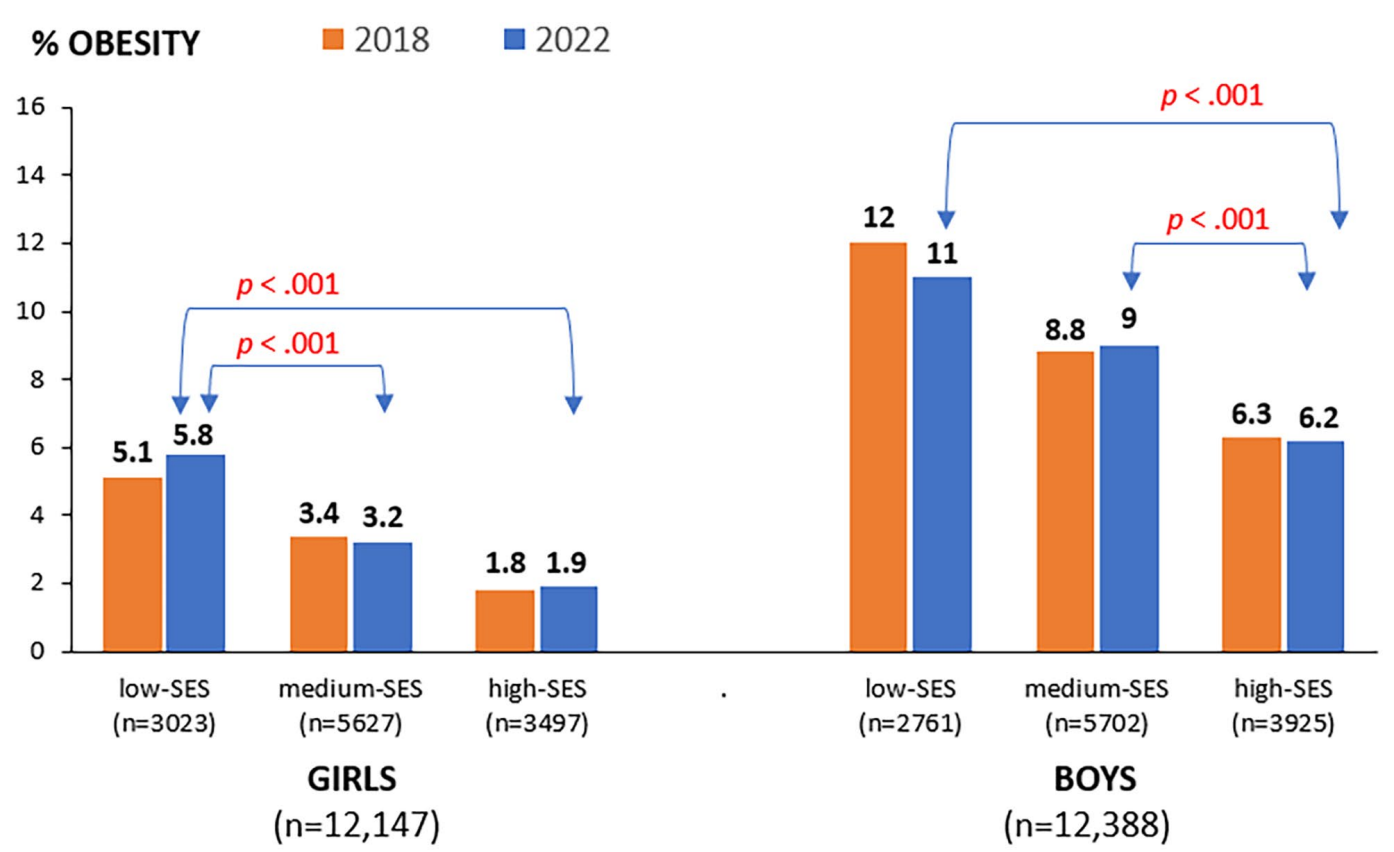
The prevalence of obesity in Czech adolescents aged 11–15 years in 2018 and 2022. *n* Number, *SES* Socioeconomic status; *p* level of statistical significance

Between 2018 and 2022, there were no significant differences using the chi-square tests in the prevalence of obesity in cohorts of girls or boys overall, and between girls or boys stratified by SES category (Table 1; Fig. 1).

In 2022, the highest prevalence of obesity was observed in adolescents from low-SES families, 11% for boys and 5.8% for girls, which is significantly (*p* < .001) higher by + 4.8% points for boys and + 3.9% points for girls than the prevalence of obesity among adolescents from high-SES families (Fig. 1). In both data collection cycles, girls from all the SES family categories reported a significantly (*p* < .001) lower prevalence of obesity than boys.

### Correlates of obesity

An overview of the obesity covariates analysed is presented in Table 2. It was confirmed that the level of SES significantly determines the incidence of obesity in Czech adolescents. Adolescents from low SES families have significantly higher odds of being obese than adolescents from medium and high SES families (Table 2).

**Table 2:** Covariates of obesity in Czech adolescents – year 2022

			95% CI
	n (%)^a^	OR	lower-upper
**SES**			
low	2760 (21.8)	Ref.	
medium	5983 (47.3)	0.77*	**0.63–0.93**
high	3899 (30.2)	**0.55‡**	**0.44–0.70**
**Gender**			
Girls	6379 (50.5)	Ref.	
Boys	6263 (49.5)	**2.98‡**	**2.48–3.58**
**Age Category**			
15 years	4011 (31.7)	Ref.	
13 years	4387 (34.7)	1.26*	**1.02–1.55**
11 years	4244 (33.6)	**1.46‡**	**1.18–1.81**
**60 min of MVPA**			
0–6 days	9863 (78.3)	Ref.	
7 days	2734 (21.6)	**0.56‡**	**0.44–0.72**
**VPA**			
< 3 times a week	5721 (45.5)	Ref.	
≥ 3 times a week	6843 (54.5)	**0.76†**	**0.64–0.91**
**Participation in organised sport**			
Inactive (no participation)	5014 (39.9)	Ref.	
Team and/or individual	7539 (60.1)	**0.71‡**	**0.60–0.85**
**Screen time**			
≥ 4 h per weekday	3077 (25.2)	Ref.	
< 4 h per weekday	9110 (74.8)	**0.58‡**	**0.45–0.74**
**Breakfast pattern**			
never	863 (6.9)	Ref.	
inconsistent	2117 (16.9)	0.77	0.56–1.07
daily on weekend days only	2038 (16.2)	0.80	0.57–1.11
always-weekend, occasionally during the week	2427 (19.3)	0.68*	**0.49–0.95**
daily	5111 (40.7)	**0.62†**	**0.46–0.85**
**Consumption of sweets**			
daily	10,052 (79.9)	Ref.	
less than daily	2522 (20.1)	**1.43†**	**1.13–1.80**
**Sleeping pattern**			
insufficient weekend + week	1899 (15.5)	Ref.	
excessive at the weekend + week	2251 (18.4)	**0.61†**	**0.46–0.83**
sufficient weekend, insufficient week	3856 (31.5)	0.89	0.70–1.12
sufficient weekend + week	4230 (34.6)	**0.71†**	**0.56–0.91**

*SES* Socioeconomic status, *CI* 95% confidence interval, *%*^*a*^ percentage of obese adolescents per independent variable, *OR* odds ratio (logistic regression) of being obese, *Ref.* Reference group, *PA* physical activity, *MVPA* moderate-to-vigorous physical activity, *VPA* vigorous physical activity, *p < .05, ^†^*p* < .005, ^‡^*p* < .001 Significant associations (*p* < .05) are in bold

Following the observed significant differences in the prevalence of obesity between adolescents from low- and high-SES families in 2022 (Fig. 1), its covariates are presented in detail separately for each category of low-SES families (Table 3). The covariates of obesity in Czech adolescents that were analysed point to significant differences in energy balance-related behaviours between the cohort of adolescents from low-SES families and their peers from medium- and high-SES families (Table 3). The odds of obesity in adolescents from low-SES families are significantly reduced by regular PA – 60 min of MVPA daily or performing VPA at the WHO’s recommended frequency of three or more times per week (Table 3).

**Table 3:** Covariates of obesity in Czech adolescents differentiated by the SES – year 2022

	Obesity								
	Low SES			Medium SES			High SES		
	%^a^	OR	95% CI lower-upper	%^a^	OR	95% CI lower-upper	%^a^	OR	95% CI lower-upper
**Gender**									
Girls	5.8	Ref.		3.2	Ref.		1.9	Ref.	
Boys	11.0	**2.22‡**	**1.60–3.09**	9.0	**3.18‡**	**2.44–4.16**	6.2	**4.08‡**	**2.62–6.34**
**Age Category**									
15 years	7.4	Ref.		6.1	Ref.		3.4	Ref.	
13 years	9.0	1.70*	**1.11–2.58**	5.7	1.26	0.93–1.72	4.2	1.66*	**1.05–2.64**
11 years	8.6	1.51*	**1.01–2.25**	6.6	1.08	0.81–1.45	5.2	1.41	0.91–2.19
**60 min of MVPA**									
0–6 days	9.3	Ref.		6.5	Ref.		4.9	Ref.	
7 days	3.8	**0.40†**	**0.22–0.71**	4.8	0.73	0.52–1.03	2.4	**0.44†**	**0.27–0.72**
**VPA**									
< 3 times a week	10.3	Ref.		7.1	Ref.		5.1	Ref.	
≥ 3 times a week	6.1	0.70*	**0.50–0.98**	5.3	0.79	0.62–1.02	3.6	0.78	0.54–1.14
**Participation in org. sport**									
Inactive (no participation)	10.1	Ref.		7.4	Ref.		5.0	Ref.	
Team and/or individual	6.7	0.73	0.53–1.03	5.3	**0.70** ^**†**^	**0.54–0.89**	3.8	0.71	0.48–1.05
**Screen time**									
≥ 4 h per weekday	9.5	Ref.		7.0	Ref.		4.8	Ref.	
< 4 h per weekday	4.7	0.60*	**0.37–0.95**	3.5	**0.61†**	**0.43–0.86**	2.2	0.50*	**0.30–0.84**
**Breakfast pattern**									
never	13.5	Ref.		8.6	Ref.		3.1	Ref.	
inconsistent	9.1	0.57*	**0.34–0.96**	7.6	0.86	0.53–1.40	5.2	1.42	0.56–3.63
daily on weekend days only	7.7	0.51*	**0.29–0.88**	6.8	0.89	0.55–1.45	5.1	1.73	0.68–4.36
always-weekend, occasionally during the week	6.6	**0.44†**	**0.25–0.78**	5.9	0.77	0.47–1.26	4.2	1.36	0.54–3.39
daily	7.7	0.54*	**0.33–0.89**	4.9	0.62*	**0.39–0.99**	3.8	1.17	0.48–2.84
**Consumption of sweets**									
daily	6.9	Ref.		4.1	Ref.		2.5	Ref.	
less than daily	8.8	1.19	0.80–1.77	6.5	1.51*	**1.07–2.14**	4.6	1.63	0.98–2.72
**Sleeping pattern**									
insufficient weekend + week	8.2	Ref.		9.1	Ref.		7.1	Ref.	
excessive weekend + week	7.8	1.09	0.62–1.92	4.3	**0.51†**	**0.33–0.79**	2.7	0.47*	**0.25–0.88**
sufficient weekend, insufficient week	11.4	1.59	0.99–2.55	6.8	0.81*	**0.58–1.12**	3.8	0.58*	**0.36–0.94**
sufficient weekend + week	6.3	0.84	0.51–1.39	5.4	0.69*	**0.49–0.98**	4.0	0.70	0.44–1.13

*SES* Socioeconomic status, *CI* 95% confidence interval, *%*^*a*^ percentage of obese adolescents per independent variable stratified by SES, *OR* odds ratio (logistic regression) of being obese in separately conducted SES analysis, *Ref.* Reference group, *PA* physical activity, *MVPA* moderate-to-vigorous physical activity, *VPA* vigorous physical activity, *p < .05, ^†^*p* < .005, ^‡^*p* < .001 Significant associations (*p* < .05) are in bold

Lower levels of daily ST significantly reduce the odds of obesity in all adolescents, regardless of their family SES. However, the covariates of adolescents’ breakfast regularity and sleep duration vary with respect to the SES category of the adolescents’ families. Skipping breakfast is most common among adolescents from low-SES families and poses a significant risk of obesity. Any form of weekly breakfast frequency among low-SES adolescents significantly reduces their odds of obesity compared to skipping breakfast (Table 3). Regression analyses comparing breakfast patterns of “never” vs. “at least one day per week” also reveal a significantly higher risk of obesity in low-SES adolescents when skipping breakfast (univariate: OR = 2.09, 95% CI = 1.37–3.18, *p* < .001; controlled for all covariates: OR = 1.20, 95% CI = 1.24–2.97, *p* < .005). This significant pattern of covariates of breakfast regularity is not evident in other categories of adolescents’ SES. Conversely, varying sleep duration is not significantly associated with the odds of obesity among adolescents from low-SES families, in contrast to adolescents from medium- and high-SES families, where insufficient sleep is significantly associated with a higher prevalence of obesity (Table 3). However, regression analysis comparing sleeping patterns of “sufficient weekend + week” vs. “other clusters” reveals significantly lower odds of obesity in low-SES adolescents (univariate: OR = 0.61, 95% CI = 0.43–0.86, *p* < .005; controlled for all covariates: OR = 0.65, 95% CI = 0.45–0.92, *p* < .05) than their counterparts from medium- and high-SES families.

## Discussion

Between 2018 and 2022, there was no significant increase in the prevalence of obesity among Czech adolescents in any of the family SES categories, and thus the assumption of an adverse effect of the COVID-19 pandemic on the increased prevalence of obesity among adolescent boys and girls from low-SES families was not confirmed. However, the adverse higher prevalence of obesity in adolescents from low-SES families compared to adolescents from medium- and high-SES families still persists. This difference in the prevalence of obesity between adolescents from low- and high-SES families is almost double for boys (or girls) – 11% versus 6.2% (triple – 5.8% versus 1.9%). A more accurate comparison of the prevalence of obesity in Czech adolescents with the prevalence of obesity in adolescents from neighbouring European countries is complicated by differences in the methodology used for measuring BMI [1] or the cumulative presentation of overweight and obesity simultaneously [16], but it seems that the prevalence of obesity in Czech adolescent boys (8.5%) is close to the overall average of obesity in adolescent boys (8.6%) in the European Union member states, while the prevalence of obesity in Czech adolescent girls (3.4%) is lower by more than 2% points than the overall average of obesity in adolescent girls (5.6%) from the European Union [1]. Among neighbouring countries, the prevalence of obesity in Czech adolescents is comparable to that of their Austrian peers, while in girls it is comparable to that of girls in Poland (3.8%) and in boys to that of boys in Portugal (8.7%) [1, 16]. Although there has been no significant increase in the prevalence of obesity in Czech adolescents over the shorter four-year follow-up period, over the longer time horizon, an annual increase in obesity of 3.8% is projected for Czech 10-19-year-olds between 2020 and 2035, with a direct negative economic impact of 3.4% of gross domestic product [39]. This projected annual increase in obesity among Czech adolescents is higher than the projected increase in obesity among adolescents from Germany (2.4%) or Austria (2.8%), but lower than that of 10-19-year-olds from Poland (4.8%), Slovakia (5.7%), or Hungary (4.2%) [39].

Analysis of the current correlates of the prevalence of obesity in Czech adolescents shows significant differences in the effect of PA, represented by both MVPA and VPA, breakfast regularity, and sleep duration on the odds of obesity in cohorts of adolescents stratified by their family’s SES. Inappropriate behaviours causing a long-term positive energy balance, which leads to an increased prevalence of obesity, appear to be more deeply embedded in the lifestyles of adolescents from low-SES families than their peers with medium and high SES. Although there was no increase in the prevalence of obesity in any of the adolescent SES categories between 2018 and 2022, it is possible that socioeconomic changes have prevented low-SES families from spending resources to encourage their children to participate in organised PA or to eat more healthily. In addition to adolescents’ own behaviours, parents’ body weight level, smoking habits, PA, and shared diet have been shown to mediate the relationship between family SES and adolescent obesity [40]. In the era of lifestyle restrictions because of the response to the COVID-19 pandemic, the shaping of children’s and adolescents’ lifestyles relied primarily on parents, and the behaviours of offspring may have mimicked those of their parents more closely than would be the case in the absence of restrictive social constraints.

There is published evidence that skipping breakfast [41, 42] and insufficient sleep [42–45] are significantly associated with childhood and adolescent obesity, but these studies did not account for the SES of adolescent families [41, 43–45] or find differences between groups of adolescents from families with different SES [42]. Of note, skipping breakfast is significantly associated with lower odds of obesity only among adolescents from low-SES families, whereas insufficient sleep is significantly associated with lower odds of obesity, in contrast, only among adolescents from medium- and high-SES families. It is possible that the parents of adolescents from low-SES families can control their offspring’s evening sleep better but not the regularity with which they eat breakfast because of early departure for work, in contrast to parents from medium- and high-SES families. Parents of adolescents from higher-SES families may typically have later bedtimes and waking-up times, and therefore may be able to better control the regularity with which their offspring eat breakfast and may be more tolerant of their offspring’s later bedtimes. However, a more detailed analysis of the association between sleep duration and sleep quality, also considering, for example, social jet lag and obesity in adolescents stratified by family SES, is the subject of a further study or studies using the 24-hour exercise behaviour of parents and their children [46].

Also of interest is the finding that less than daily consumption of sweets is associated with higher odds of obesity virtually for all SES categories of adolescents. This finding is in line with previous studies using the HBSC data [14, 47]. The most plausible explanation seems to be that those who face troubles with their weight status already at this age, regulate (or have it regulated by their families) their energy intake to greater extent, including constraints concerning consumption of sweets [48]. Non-overweight adolescents and especially those with high energy expenditure, e.g., in sports, do not feel a need to impose any such restrictions. Moreover, non-obese adolescents are more likely to participate in vigorous physical activity and organised physical activity than their obese peers and to supplement the immediate exertion/exhaustion resulting from these types of physical activity with sweets.

Building on the findings of a mediating relationship between parental behavioural characteristics, family SES, and adolescent body fat [40], the finding that adolescents from low-SES families are significantly more likely to be obese at the age of 11 and 13 years compared with 15 years is of great concern. Thus, soon, we can expect the most significant increase in obesity in the category of adolescents from low-SES families. Moreover, the finding of a higher likelihood of obesity in the younger categories of adolescents from low-SES families is reinforced when compared with the previous situation in 2002–2018 [14].

### Strengths and limitations

The strengths of the study include the ability to generalise the results and conclusions to the entire population of Czech adolescents aged 11–15 years because of the nationally representative sample of participants and the uniform methodology used in the same time frame of data collection. There was strict adherence to an international research protocol using a standardised questionnaire, which allows for subsequent international comparison. The main methodological limitation of this study is the possibility of response bias resulting from the participants’ subjective responses; however, this possibility was avoided by including questions and response items that met strict validity and reliability requirements [5]. Furthermore, the cross-sectional design of the study does not allow for causal interpretation of the results on the relationship between behavioural characteristics related to individuals’ energy balance and the prevalence of obesity; however, genetic factors alone do not explain the global obesity epidemic [3], and therefore socioeconomic factors must also be considered [49].

## Conclusions

There were no significant changes in the prevalence of obesity among adolescent girls and boys between 2018 and 2022, but the highest prevalence of obesity among adolescents from low-SES families compared to adolescents from medium- or high-SES families persisted. Obesity, because of a long-term positive energy balance mediated by imbalanced behaviour, is most pronounced in adolescents from low-SES families. It was confirmed that significantly lower odds of obesity in adolescents from low-SES families are associated with regular participation in recommended PA, shorter ST duration, and not skipping regular breakfast. Insufficient sleep is associated with significantly higher odds of obesity in adolescents from families with medium and high SES.

## Data Availability

The datasets that were generated and analysed during the current study are not publicly available because of the rules for funded projects but are available from the corresponding author ES upon reasonable request.

## List of Abbreviations

BMI: Body Mass Index
CI: Confidence interval
FAS: Family Affluence Scale
HBSC: Health Behaviour in School-aged Children
ICC: Intra-class correlation
M: Mean
MVPA: Moderate-to-vigorous physical activity
OR: Odds ratio
PA: Physical activity
PC: Computer use
Ref.: Reference group
SES: Socioeconomic status
SPSS: Statistical Package for the Social Sciences
ST: Screen time
TV: Television viewing
VPA: Vigorous physical activity
WHO: World Health Organization
